# Omics analysis of Alzheimer’s disease stratified by the microglial polygenic effect

**DOI:** 10.1002/alz.088489

**Published:** 2025-01-03

**Authors:** Masataka Kikuchi, Akinori Miyashita, Yu Hirota, Norikazu Hara, Mai Hasegawa, Yasufumi Sakakibara, Michiko Sekiya, Yuko Saito, Shigeo Murayama, Koichi M. Iijima, Takeshi Ikeuchi

**Affiliations:** ^1^ Department of Computational Biology and Medical Sciences, Graduate School of Frontier Science, The University of Tokyo, Chiba Japan; ^2^ Brain Research Institute, Niigata University, Niigata, Niigata Japan; ^3^ National Center for Geriatrics and Gerontology, Obu Japan; ^4^ Tokyo Metropolitan Geriatric Hospital and Institute of Gerontology, Tokyo Japan; ^5^ Brain Bank for Neurodevelopmental, Neurological and Psychiatric Disorders, Osaka University, Osaka Japan

## Abstract

**Background:**

Recent single‐cell omics analyses have revealed that microglia change into reactive microglia when Aβ accumulates in the brain and exhibit Aβ phagocytosis. However, reactive microglia are less likely to be induced in TREM2 mutation carriers. This microglia‐centred pathological mechanism may be considered one of the pathologies of AD. However, a single gene mutation, such as a TREM2 mutation, can rarely explain the pathology of AD. Therefore, polygenic effects involving multiple gene mutations have been proposed. In this study, we propose a polygenic risk score (PRS) to quantify the microglial polygenic effect. We stratified patient groups by this PRS and examined differences in pathogenesis based on omics analysis using postmortem brains.

**Methods:**

Whole‐genome sequencing and bulk brain tissue RNA‐seq of the frontal cortex were performed using postmortem brain samples from 100 subjects with Braak senile plaque (SP) and neurofibrillary tangle (NFT) staging information. In addition, single‐nuclei RNA‐seq (snRNA‐seq) was performed on 15 of these subjects (healthy subjects: 8 subjects, AD patients: 7 subjects).

**Result:**

We divided AD patients into low and high groups by microglial PRS and performed bulk brain tissue RNA‐seq analysis. We identified 112 differentially expressed genes and found that those genes were related to autophagy and inflammation. To further validate these findings at the single‐cell level, we identified cell clusters containing microglia based on snRNA‐seq. Gene expression analysis of microglia in 3 AD patients with low PRS and 4 AD patients with high PRS suggested altered transcriptional profiles of immune cells and phagocytosis. In addition we observed different microglial subtypes in different PRS.

**Conclusion:**

The stratification analysis of AD by PRS suggests that microglial reactivity differs among AD patient groups. Future detailed microglial observations in postmortem brains from AD patients with low PRS and those with high PRS will further elaborate our results.

